# To Sleep, to Strive, or Both: How Best to Optimize Memory

**DOI:** 10.1371/journal.pone.0021737

**Published:** 2011-07-20

**Authors:** Matthew A. Tucker, Sunny X. Tang, Amaka Uzoh, Alexandra Morgan, Robert Stickgold

**Affiliations:** 1 Department of Psychiatry, Harvard Medical School, Boston, Massachusetts, United States of America; 2 Department of Psychology, Harvard University, Cambridge, Massachusetts, United States of America; 3 Center for Sleep and Cognition, Boston, Massachusetts, United States of America; University of Alberta, Canada

## Abstract

While numerous studies have shown that a night of sleep profits memory relative to wake, we still have little understanding about what factors mediate this effect of sleep. A clear understanding of the dynamics of this effect of sleep beyond the initial night of sleep is also lacking. Here, we examined the effect of extrinsic rewards on sleep-dependent declarative memory processing across 12 and 24 hr training-retest intervals. Subjects were either paid based on their performance at retest ($1 for each correct answer), or received a flat fee for participation. After a 12 hr interval we observed pronounced benefits of both sleep and reward on memory. Over an extended 24 hr interval we found 1) that an initial night of sleep partially protects memories from subsequent deterioration during wake, and 2) that sleep blocks further deterioration, and may even have a restorative effect on memory, when it follows a full day of wake. Interestingly, the benefit imparted to rewarded (relative to unrewarded) stimuli was equal for sleep and wake subjects, suggesting that the sleeping brain may not differentially process rewarded information, relative to wake. However, looking at the overall impact of sleep relative to reward in this protocol, it was apparent that sleep both imparted a stronger mnemonic boost than reward, and provided a benefit to memory regardless of whether it occurred in the first or the second 12 hrs following task training.

## Introduction

During our day-to-day lives, we encode enormous amounts of fact-based information, some of which is crucial to intellectual, academic, and career success, but much of which is not. For example, a student may consider information relevant to an upcoming test to be important, but may deem other information as personally irrelevant (e.g., the name of an unimportant character in a forgettable movie).

What information does an individual deem personally relevant? Research clearly demonstrates that attaching a monetary reward to a stimulus during encoding is one means of enhancing information processing [1], [2]. Even minimal performance-based rewards (monetary rewards as little as $1) can have a significant impact on subsequent recall of information [3]. This behavioral enhancement is echoed in findings from human brain imaging studies showing that increased activation of reward-relevant brain circuitry following presentation of reward-contingent stimuli correlates with greater retention of the rewarded information [4], [5].

While it is well established that extrinsic rewards can enhance memory, studies that examine the effect of reward on memory typically are conducted over brief periods of wake (typically less than two hours) or after a one week delay [2], without addressing the potential benefits of post-acquisition sleep, a physiological state known to benefit memory for most forms of information [6], [7]. If sleep benefits memory when subjects are paid merely to participate (*i.e.*, not based on performance), will reward further increase the benefit produced by sleep? Thus far, only one study, using a procedural typing task, has examined the effects of combined sleep and reward [8]. Following training on two 5-digit sequences, subjects were told that they would be rewarded for better performance on just one of the sequences at retest. Overall, subjects who slept between training and retest performed better (typed faster) than those who remained awake. Importantly, however, sleep subjects demonstrated greater overnight gains in speed for the rewarded sequence than for the unrewarded one, gains which were not observed following a day of wakefulness. The current study takes a different approach by instead examining declarative memory (visual paired associates) and by informing subjects *prior* to training that performance would be rewarded at retest. This design provides the opportunity to examine the impact of reward on encoding as well as post-encoding processing of declarative memory (for example, see [1]), but also to assess this reward-related impact as it occurs over periods of sleep and wake. The design has the added advantage of simulating many real world situations (*e.g.*, students take classes knowing from the outset that they will be working to achieve a good grade) and by providing a powerful incentive to learn well (*i.e.*, the potential to quadruple their payment by getting all items correct at retest).

**Figure 1:**
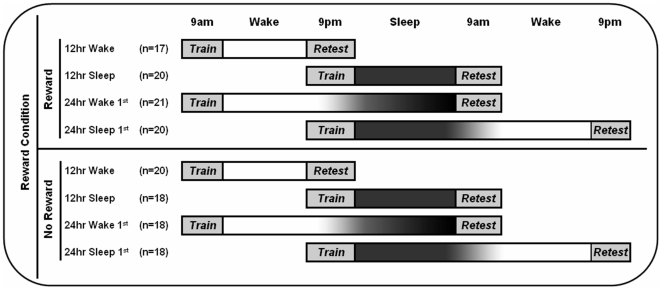
Study design.

In this study, we also examined memory performance when sleep occurred in either the first or second half of the 24 hr period (see Figure 1). This analysis is critical because, even though recall of declarative (fact-based) information is superior following a night of sleep compared to an equal period of daytime wake [7], [9], it is unclear, using a 12 hr training-retest interval, whether sleep has to closely follow training or whether it can benefit memory when it occurs more than 12 hrs after training (*i.e.*, after a full day of wake). One study thus far reports that performance on a spatial memory task (face-location associations), at 24 hr retest, benefits from sleep when sleep closely follows training, but not when 12 hrs of wake are interposed between training and sleep [10]. Another study has demonstrated the same effect for vocabulary learning [11]. However, a third study, using a word pair learning task with children 9–12 yrs of age, showed similar sleep benefits regardless of whether sleep occurred during the first or second 12 hrs of the training-retest interval [12].

Employing the standard 12 hr training-retest interval it is also unclear whether sleep can stabilize memory, preventing degradation across a subsequent waking interval in which non-specific interference effects would be expected [13]. Interestingly, the spatial memory study described above [10] found that memory at 24 hr retest in subjects that slept during the first 12 hrs was not significantly worse than recall after a 12 hr interval filled with sleep, suggesting that an initial night of sleep helps protect the memory from the interference one would expect to occur during the subsequent wake period.

Here we hypothesized that sleep would benefit visual declarative memory more than wake regardless of when it occurs in the 24 hr interval (*i.e.*, in the first or the second 12 hr interval following training), and that monetary reward would benefit memory at 12 hr retest and 24 hrs following training. Based on the findings of the one related study described above [8], we also expected a possible interaction between sleep and reward, such that sleep, compared to wake, would impart a greater benefit for rewarded than unrewarded information.

## Method

### Ethics Statement

Informed written consent was obtained from all participants and the study was approved by the Beth Israel Deaconess Medical Center Institutional Review Board.

### Subjects

Subjects were 152 Harvard undergraduates (62 males, 90 females, mean age 20.0±1.7 yrs [SD]) in good health and free of medications that affect sleep or cognition. All subjects were instructed to abstain from alcohol and caffeine 24 hrs prior to and during the study. The study protocol, presented in Figure 1, includes 8 groups of subjects and represents a condition (Sleep v. Wake)×reward (Reward v. No-Reward)×time (12 hr v. 24 hr) factorial design.

### Visual Paired Associates

**Figure 2:**
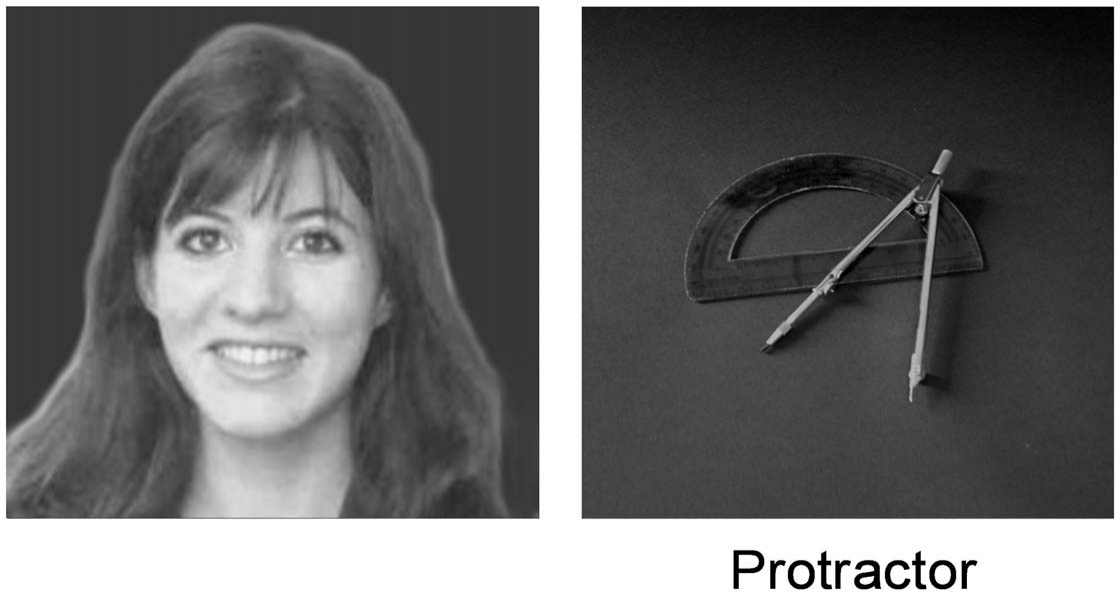
Example of a Visual Paired Associate.

The visual paired associates (VPA) task consists of 30 black-and-white face-object pairs with the name of the object displayed under the object (Figure 2). The photos were equated for contrast and brightness. Each of the 30 pairs was presented for 5 seconds. After presentation of the picture pairs, subjects were quizzed on the pairs – the 30 faces were presented in random order, and subjects attempted to recall the object that was paired with each face, typing the answer in a text box next to the face. After each response, the correct answer was presented for 4 seconds. If the subject entered a correct answer, that pair was not presented again. If, after the first presentation of all 30 pairs, the subject missed more than 6 items (20%), all missed items from the previous trial were re-presented (in random order) until 24 of the 30 pairs had been correctly recalled (criterion of 80% correct). Subjects were then given an immediate cued recall test to assess their memory for the pairs, with all 30 cues presented in a newly randomized order, but without the correct answers being presented after each response. At retest, either 12 or 24 hrs later, the cued recall test was repeated, again without feedback.

### Procedure

Subjects arrived at a Harvard University computer laboratory at 9am or 9pm. They provided written consent and completed visual analog scales, asking: 1) “How would you describe your ability to concentrate right now?” and, 2) “How refreshed do you feel right now?” They also completed the Epworth Sleepiness Scale [14], which assessed how likely they were to fall asleep in a number of common situations (*e.g.*, while reading a book), as well as the Stanford Sleepiness Scale [15], in which they rated their level of sleepiness just prior to VPA training. A questionnaire assessing subjects' habitual sleep patterns was then completed, as well as a retrospective sleep log in which subjects reported bedtime, wake time, total sleep time, and sleep quality for the three nights prior to the study.

After completing the forms, subjects in the Reward condition were informed prior to training that, in addition to the $10 they would be paid for participating in the study, they would also be paid $1 for each correct answer they gave at retest (up to $40 total). The No-Reward subjects were instead informed that they would receive $30 for their participation. This amount was determined based on pilot data indicating that rewarded subjects earned, on average, $30 for their performance, ensuring that all subjects, regardless of condition, would be paid a similar amount for participation. All subjects then trained on the VPA task and performed the immediate cued recall test.

When subjects returned for retest, either 12 or 24 hrs later, they again completed the alertness and sleepiness scales as well as two visual analog scale questions: 1) “How motivated were you to do well on this task?”, and 2) “How much did you think about this task before the retest session?” An additional one-night sleep log was completed by subjects who slept between the training and retest sessions.

## Results

### Sleep Log and Sleepiness/Alertness Data

All groups reported similar sleep log data and subjective alertness at training and retest. Average bedtime prior to the training session was 1:15am±1.4 hrs (SD), and total sleep time averaged 7.1±1.5 hrs, which was essentially the same as their reported habitual sleep duration (7.2 hrs). Amount of sleep obtained the night before training was similar across Sleep and Wake conditions (12 hr Wake: 6.9±1.2 hrs, 12 hr Sleep: 7.1±2.0 hrs, 24 hr Sleep-First: 7.5±1.4 hrs, 24 hr Wake-First: 7.1±1.2 hrs, One-way ANOVA, F_3,147_ = 1.14, p = .33). The 24 hr Wake-First groups went to bed at 1:33am±1.7 hrs, approximately 16 hrs following training, and slept an average of 6.8±1.4 hrs prior to retest at 9am. The 24 hr Sleep-First groups slept an average of 7.5±1.3 hrs following training at 9pm, and awoke at 8:52am±2.5 hrs, approximately 12 hrs before retest at 9pm.

Epworth Sleepiness Scale (ESS) scores were similar across experimental groups (range: 7.8–10.6, one-way ANOVA, F_7,144_ = 1.15, p = .33), as was subjective sleepiness reported on the Stanford Sleepiness Scale (SSS) for subjects that trained in the morning (2.6±0.1) v. evening (2.7±0.1) (t_149_ = .53, p = .60). There was no difference between groups at training on VAS-reported “ability to concentrate” (p = .45) or for how “refreshed” subjects felt (p = .13) prior to training.

### Training Performance

Training performance, measured as the number of trials to reach criterion at training and the number of correct answers on the immediate cued recall test, did not differ between the 12 hr groups that trained in the evening (12 hr Sleep) v. morning (12 hr Wake) (trials to criterion: Sleep: 3.8±0.2 [mean±SEM], Wake: 3.6±0.2, t_72_ = .63, p = .53; number correct: Sleep: 22.1±0.7, Wake: 23.1±0.4, t_73_ = 1.33, p = .19). There was also no difference between the 24 hr Sleep-First and Wake-First groups on these variables (trials to criterion: Sleep-First: 3.9±0.2, Wake-First: 3.8±0.3, t_75_ = .28, p = .78; number correct: Sleep-First: 21.4±0.7, Wake-First: 22.5±0.7, t_75_ = 1.17, p = .25). The one-way ANOVAs comparing training performance across all groups were non-significant (trials to criterion: p = .78, number correct: p = .26, LSD comparisons, all ps>.05).

### The Effect of Sleep and Reward on Memory

**Figure 3:**
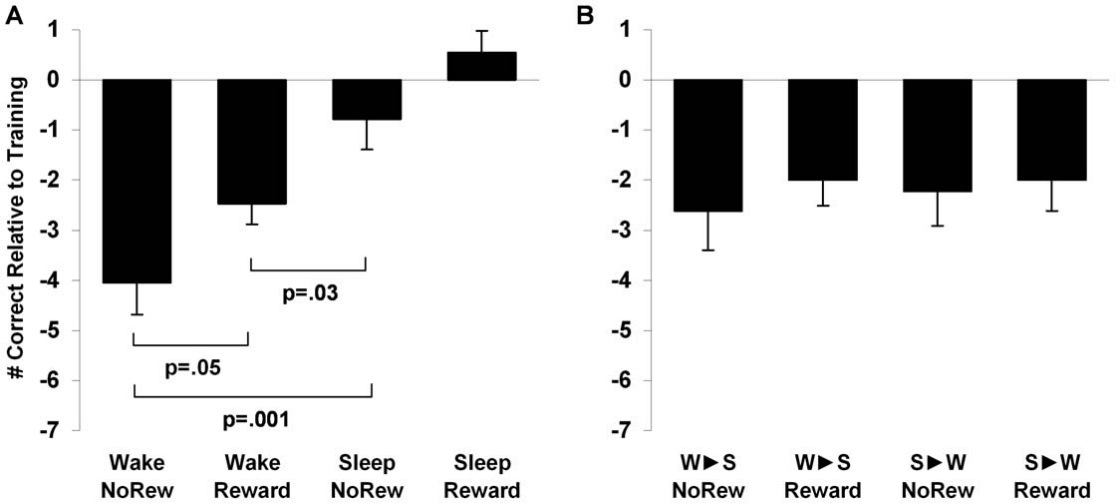
Performance data for all groups. A. 12 hr groups. B. 24 hr groups. (means±SEMs).

**Figure 4:**
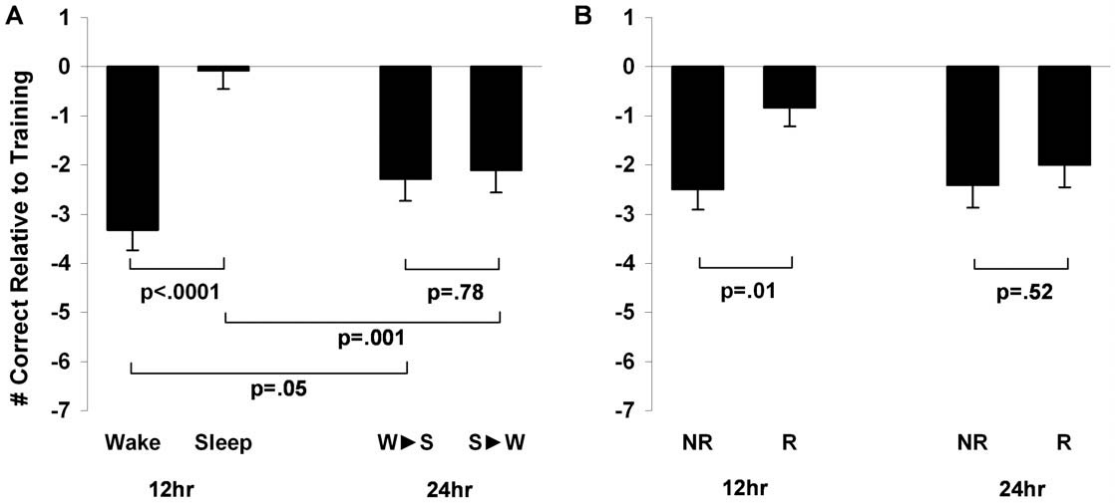
Sleep and Reward effects. A. Sleep v. Wake performance at 12 and 24 hrs. B. Reward v. No-Reward performance at 12 and 24 hrs. Bars represent change in recall collapsed across reward condition (means±SEMs).

Data for the individual 12 hr groups are presented in Figure 3A. We observed independent benefits from Sleep and Reward, such that subjects in the 12 hr Sleep+Reward condition performed best, actually improving their performance by 0.6 picture pairs (+2.4%) at retest (p = .21), while the 12 hr Wake+No Reward group performed worst, forgetting 4.1 pairs (17.0%; p<.0001; Figure 3A). Overall, there was a robust memory advantage at 12 hr retest for subjects who slept, with the 2-way ANOVA of condition (Sleep v. Wake)×reward (Reward v. No-Reward) revealing a highly significant main effect of condition (F_1,73_ = 34.09, p<.0001, η^2^_p_ = .32; Figure 4A, left). Specifically, wake subjects forgot 3.3±0.4 (14.2%) pairs (t_36_ = 8.14, p<.0001), while sleep subjects demonstrated highly preserved memory, forgetting only 0.1±0.4 (0.4%) picture pairs (t_37_ = .21, p = .84).

Anticipated monetary reward also had a distinct impact on performance. While Reward subjects reported greater motivation (p = .03) and thinking more about the task (p = .001), Reward and No-Reward subjects performed similarly at training (trials to criterion: Reward: 3.8±0.2, No-Reward: 3.7±0.2, t_149_ = .50, p = .62; number correct: Reward: 22.5±0.4, No-Reward: 22.0±0.5, t_150_ = 0.88, p = .38), indicating that prior knowledge of the performance-based reward did not influence training performance. However, at 12 hr retest, there was a significant main effect of reward (F_1,73_ = 6.65, p = .01, η^2^_p_ = .08; Figure 4B, left), such that Reward subjects showed only minimal forgetting of 0.8±0.4 (3.7%) pairs, while the No-Reward subjects forgot 2.5±0.5 (10.6%) pairs. When retest was delayed until 24 hrs, the difference between Reward and No-Reward subjects disappeared, with rewarded subjects now forgetting 2.0±0.4, and non-rewarded subjects forgetting 2.4±0.5 pairs (t_75_ = .43, p = .52; Figure 4B, right) (reward×time (12 hr v. 24 hr) interaction (F_1,148_ = 1.88, p = .17, η^2^_p_ = .01)).

**Figure 5:**
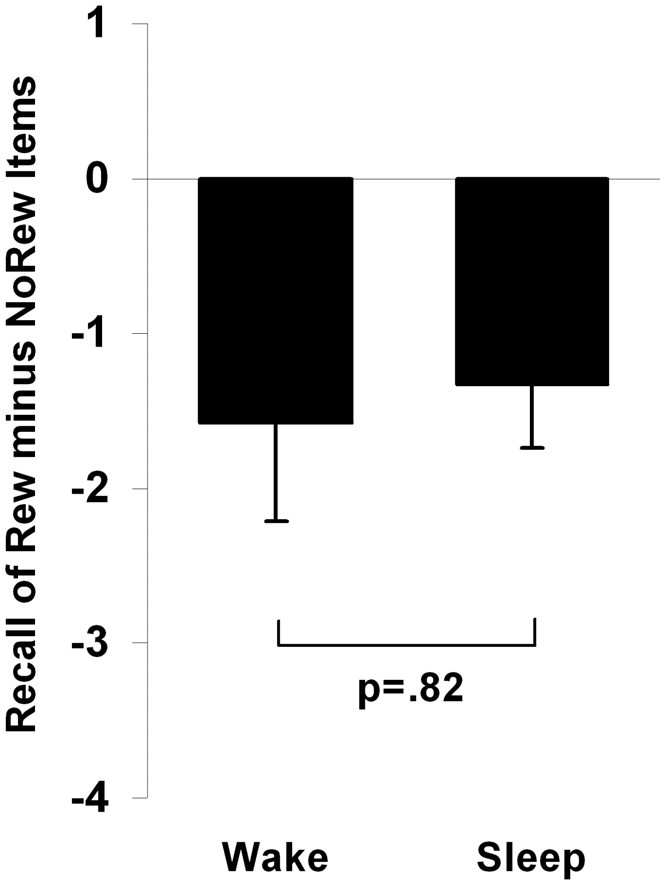
Difference between recall of Rewarded and Unrewarded stimuli in the 12 hr Wake and Sleep subjects indicating the non-significant interaction between sleep and reward. Bars represent change in recall from initial testing (means±SEMs).

Evaluating the relationship between sleep and reward at 12-hr retest, we found a non-significant interaction (F_1,71_ = .06, p = .82, η^2^_p_ = .001), suggesting that sleep, compared to wake, does not preferentially process rewarded relative to unrewarded information (*i.e.*, the difference in recall between rewarded and non-rewarded information did not differ between sleep and wake subjects; Figure 5).

Comparing the independent contributions of Sleep and Reward to memory, we found that the effect size of the sleep benefit (η^2^_p_ = .32) was four times that of reward (η^2^_p_ = .08), and that the difference between the Sleep effect and Reward effect was statistically significant (Fisher's Z test, p = .04). Looking at individual group differences, this memory benefit of sleep compared to reward becomes even clearer. Figure 3A shows that the benefit of sleep alone (Sleep+No-Reward vs. Wake+No-Reward; difference = 3.3±0.9 pairs, p = .001) was more than twice that of reward alone (Wake+Reward vs. Wake+No-Reward; difference = 1.6±0.8 pairs, p = .05). Indeed, wake subjects who knew that their payment depended on how well they learned the picture pairs (Wake+Reward subjects) recalled significantly fewer pairs than subjects who merely slept (Sleep+No-Reward), with no potential reward for better performance (t_33_ = 2.26, p = .03).

### The Effect of Sleep on Memory Across the 24 hr Interval

Comparisons of performance in the individual 24 hr conditions revealed no significant group differences (One-way ANOVA, F_3,73_ = .20, p = .90, LSD comparisons, all ps>.50; Figure 3B). However, when we collapsed across reward condition, we were able to examine the beneficial effect of sleep when it occurred during the first 12 and second 12 hrs of the 24 hr training-retest interval (Figure 4A). When comparing the 12 hr Sleep group to the 24 hr Sleep-First group (those who slept during the first 12 post-training hours, but were awake during the following 12 hrs), we observed a significant drop in performance following the second 12 hr interval containing wake (t_74_ = 3.43, p = .001). However, this recall 24 hrs after training was still significantly better than in the 12 hr Wake group (t_73_ = 2.00, p = .05), indicating that a night of sleep offered partial protection against subsequent memory deterioration during wake.

When comparing the 12 hr Wake subjects to the 24 hr Wake-First subjects (those who were awake during the first 12 hr, and slept during the second 12 hr), the inverse was true. While a full day of wakefulness had a pronounced deleterious effect on recall that evening, a subsequent night of sleep not only prevented further memory deterioration, but may even have had a restorative effect on memory, elevating recall to levels numerically above those observed in the 12 hr Wake group (p = .09). The beneficial effect of sleep during the first and second 12 hrs is further supported by the significant sleep×time (12 hr v. 24 hr) interaction (F_1,148_ = 13.07, p = .0004).

## Discussion

### Evaluating the Effect of Sleep and Reward on Memory

The results reported here clearly indicate that sleep provides a dramatic benefit for visual declarative memory across 12 hr and 24 hr intervals. In addition to the pronounced benefit of sleep, we observed a robust effect of reward across the initial 12 hrs, not only in terms of amount of information recalled, but also in subjective reports of motivation: subjects reported thinking more about the task between training and retest, and reported being more motivated to do well on the task than non-rewarded subjects. However, sleep (compared to wake) did not appear to provide a larger boost for rewarded information than for unrewarded information. In fact, the difference between recall of rewarded and unrewarded stimuli was almost identical in the Wake and Sleep groups. What this may suggest is that the activation of reward-relevant brain structures, such as the ventral striatum, known to occur during encoding [4], [5], does not prime the brain for augmented sleep-dependent memory processing. This is interesting in light of other studies that have shown a preferential benefit of sleep for information with strong emotional valence [16], [17], which is known to produce heightened activity in the amygdala during encoding [18], and which leads to strengthening of relevant network connectivity following sleep, for example, between amygdala and ventromedial prefrontal cortex [19].

Framing this difference in findings in evolutionary terms, it seems likely that emotional stimuli (and experiences) are preferentially processed during sleep because they are of inherently greater survival value than most forms of extrinsic reward (*e.g.*, monetary reward). On the other hand, it is noteworthy that one study using a motor memory task (typing of 5-digit number sequences) did find that sleep benefited rewarded memories more than unrewarded ones [8]. While there were differences in study design between that study and the current one, it is plausible that sleep-dependent processing of certain types of memory (*e.g.*, motor memory) are heightened by reward anticipation, while others (*e.g.*, declarative memory) are not. Future studies will be necessary to further characterize the dynamics of reward-modulated sleep-dependent memory processing across memory domains, and under differing reward contingencies. Indeed, it may be that extrinsic rewards do modulate the effect of sleep on declarative memory. In the current study, even though the sleep by reward interaction was non-significant, the sleep groups performed so well on the task (virtually maintaining their performance from training to 12 hr retest), that they may have been performing near ceiling, damping the reward-based differences in performance in the sleep groups. It may be that subtly adjusted declarative memory testing regimens would lead to greater sleep-dependent memory benefits for rewarded information.

Even though no interaction between sleep and reward was found, it is noteworthy that the magnitude of the sleep effect was greater than that of reward, with a sleep effect size that was four times greater than that for reward. This difference is further illustrated by comparing subjects who slept but did not expect to be rewarded to subjects who did anticipate a monetary reward but did not sleep. In this instance, the Sleep-No Reward subjects benefited more than Wake subjects who had the potential to quadruple ($10 vs. $40) their payment. Even when highly motivated to perform well, wake subjects simply could not reach the level of recall attained by those who merely obtained a night of sleep prior to retest. Sleep provided a boost to memory that could not be compensated for by simply “trying harder”.

It remains possible that the reward-related benefits were not entirely consolidation effects, but might also have resulted from differences in encoding, as subjects in the reward condition were informed of the reward prior to initial task training. We cannot rule out the possibility that rewarded subjects encoded the items differently despite performing similarly to the unrewarded subjects during training and immediate testing. It is also possible that subjects in the reward condition attempted to mentally rehearse the stimuli prior to retest, although the use of images (faces and objects), as opposed to verbal stimuli, would have made this extremely difficult.

### The Effect of Sleep on Memory Across the 24 hr Interval

There is a large literature demonstrating that performance on declarative memory tasks is superior following sleep as opposed to wakefulness, whether sleep comes in the form of a full night of sleep or even a daytime nap [6]. However, what is less clear is whether the superior memory performance that follows a period of sleep is due to a beneficial change to the memory during sleep or simply to the absence of non-specific deleterious effects of wakefulness [13]. The results from the 24 hr retest condition in the current study provide evidence supporting an active role for sleep. Significantly more forgetting was seen after 12 hr of daytime wake than after 24 hr that began with a night of sleep and ended with more than 12 hr of daytime wake. This strongly argues that sleep at least partially stabilized the memories, reducing the negative impact of daytime wakefulness. This finding corroborates a recent report of a similar stabilization using a spatial face-location task [10].

A second question addressed by the 24 hr retest condition is for how long after encoding sleep continues to benefit memories. When sleep occurred during the second 12 hr interval, beginning, on average 16 hrs after training, recall at 24 hr retest was no worse than after just 12 hrs of wake, actually showing a trend (p = .09) toward improving across the second 12 hrs with sleep. It is unclear whether this finding simply reflects a prevention of further deterioration of the memory trace that would be expected to occur over time, or whether it is evidence of a restorative effect of sleep on memory. Evidence of restorative effects of sleep have been reported for nondeclarative, procedural learning [20], and for declarative memory when retroactive interference is induced after encoding, but before sleep [21]. Interestingly, this restorative effect was not observed in one study [10], which found continued memory decline over the second 12 hr interval filled with sleep.

In summary, our findings confirm the active role of sleep in enhancing recently-encoded memories, and lend support to the evolving theory that the unique neuromodulatory and electrophysiological characteristics of sleep, including sleep spindles [22], [23], hippocampal sharp-wave ripples [24], [25], and reduced acetylcholine levels during slow wave sleep [26], are ideally suited for such memory processing. Not only does sleep provide a dramatic boost to memory over the short term, it appears to play an important role beyond the first 12 hrs, partially protecting the memory from subsequent waking interference, and continuing to benefit memory even when sleep occurs up to 16 hrs after initial encoding. Finally, while sleep was not found to be a preferred brain state for the processing of reward-based information, we find it remarkable that sleep nevertheless provided a stronger and more long-lasting benefit to memory than a cash incentive for better performance. In a society that places much emphasis on the power of extrinsic rewards to promote achievement, it might be prudent to reconsider the benefits of a good night of sleep.
